# The Systemic Immune-Inflammation Index (SII) and coronary artery lesions in Kawasaki disease

**DOI:** 10.1007/s10238-023-01265-0

**Published:** 2024-01-17

**Authors:** Tiantuo Huang, Qi Peng, Yiyue Zhang, Zaifu Zhu, Xiaochen Fan

**Affiliations:** Department of Pediatrics, The First Affiliated Hospital of Anhui Medical University, No.218 Ji-Xi Road, Hefei, Anhui Province China

**Keywords:** Kawasaki disease, The Systemic Immune-Inflammation Index (SII), Coronary artery lesions, Risk factors

## Abstract

Coronary artery lesions (CALs) are the most common complications of Kawasaki disease (KD) and play a crucial role in determining the prognosis of the disease. Consequently, the early identification of children with KD who are at risk of developing coronary artery damage is vitally important. We sought to investigate the relationship between the Systemic Immune-Inflammation Index (SII) and CALs in patients with KD and to assess its predictive value. We carried out a retrospective review and analysis of medical records for KD patients treated at the First Affiliated Hospital of Anhui Medical University between January 2017 and January 2023. We utilized single-variable tests, binary logistic regression analysis, ROC curve analysis, restricted cubic spline tests, and curve fitting to evaluate the association between SII and CALs. In our study, 364 patients were included, with 63 (17.3%) presenting with CALs at the time of admission. The binary logistic regression analysis indicated that SII was a significant risk factor for CALs at admission, evident in both unadjusted and models adjusted for confounders. The ROC curve analysis revealed an AUC (Area Under the Curve) value of 0.789 (95%CI 0.723–0.855, *P* < 0.001) for SII's predictive ability regarding CALs at admission. A consistent positive linear relationship between SII and the risk of CALs at admission was observed in both the raw and adjusted models. Our research findings suggest that SII serves as a risk factor for CALs and can be used as an auxiliary laboratory biomarker for predicting CALs.

## Introduction

Kawasaki disease (KD) is a form of systemic vasculitis that predominantly affects children under the age of 5 [1]. It has now emerged as the leading cause of acquired heart disease in developed nations^1^. The most severe and prevalent complication of KD are coronary artery lesions (CALs), manifesting in approximately 25% of untreated individuals [2]. Current guidelines suggest that prompt administration of intravenous immunoglobulin (IVIG) after diagnosis significantly reduces the risk of CALs [1]. Therefore, the timely identification and treatment of children susceptible to CALs are imperative.

Over the past several decades, extensive research efforts have been devoted to discerning the determinants of CALs. In addition to well-established factors such as male gender, delayed treatment, and resistance to IVIG, which are widely acknowledged as primary risk factors [3–5], laboratory parameters like low hemoglobin, reduced serum albumin, and diminished serum sodium [6, 7]. Nevertheless, there remains a subset of KD patients who develop CALs in the absence of the aforementioned factors.

The precise etiology of KD remains uncertain. Nonetheless, accumulated evidence from genetics, immunology, and experimental data suggests that Kawasaki disease arises from intricate interactions between innate immune responses to one or multiple antigens, followed by subsequent acquired immune reactions [8]. The Systemic Immune-Inflammatory Index (SII), an innovative inflammatory and immune marker derived from platelets, neutrophils, and lymphocytes (SII = platelets × neutrophils/lymphocytes), was initially introduced by Hu et al. [9]. This marker has demonstrated a correlation with the severity and prognosis in both cancer patients and individuals with inflammatory diseases [10–12]. Recent research has also associated SII with the onset of cardiovascular diseases [13, 14]. Given the link between KD and its role as a risk factor for cardiovascular diseases, especially those compromising coronary artery integrity, we hypothesize that SII may be associated with the emergence of CALs. Consequently, the primary aim of this study is to assess the correlation between SII and the presence of CALs at the time of admission.

## Methods

### Participants

Utilizing a retrospective methodological framework, we gathered data from pediatric patients diagnosed with KD, which includes both Complete KD and Incomplete KD (IKD). These patients were treated at the First Affiliated Hospital of Anhui Medical University from January 2017 to January 2023. Given the retrospective nature of our study, it was not feasible to obtain written informed consent from participants. The research was approved by the ethics review boards (Ethical Review Number:Quick—PJ 2023-12-52).The diagnostic parameters were based on the 2017 AHA guidelines. These guidelines necessitate that patients exhibit a fever lasting at least five days, accompanied by a minimum of four from the subsequent five primary clinical indicators: 1. Cutaneous rashes;2. Bilateral conjunctival hyperemia;3. Changes in oral mucosal conditions;4. Alterations in the extremities;5. Cervical lymphadenopathy, typically unilateral [1]. For patients displaying echocardiographic evidence of coronary impairment, including coronary dilation or aneurysms, a definitive diagnosis necessitates only four of the primary clinical indications. A diagnosis of IKD is confirmed when patients exhibit two or three consistent clinical features, while simultaneously ruling out other diseases with similar symptoms. The exclusion criteria include: pediatric patients who received IVIG or corticosteroids at external medical facilities; patients discharged automatically for certain reasons; individuals with inherent cardiovascular conditions, oncological diseases, hematological disorders, and similar conditions; patients had recurrence of KD; and patients lacking complete medical records. All qualifying pediatric participants, consistent with the research demographic criteria, were administered IVIG and Aspirin therapy upon admission.

**Fig. 1:**
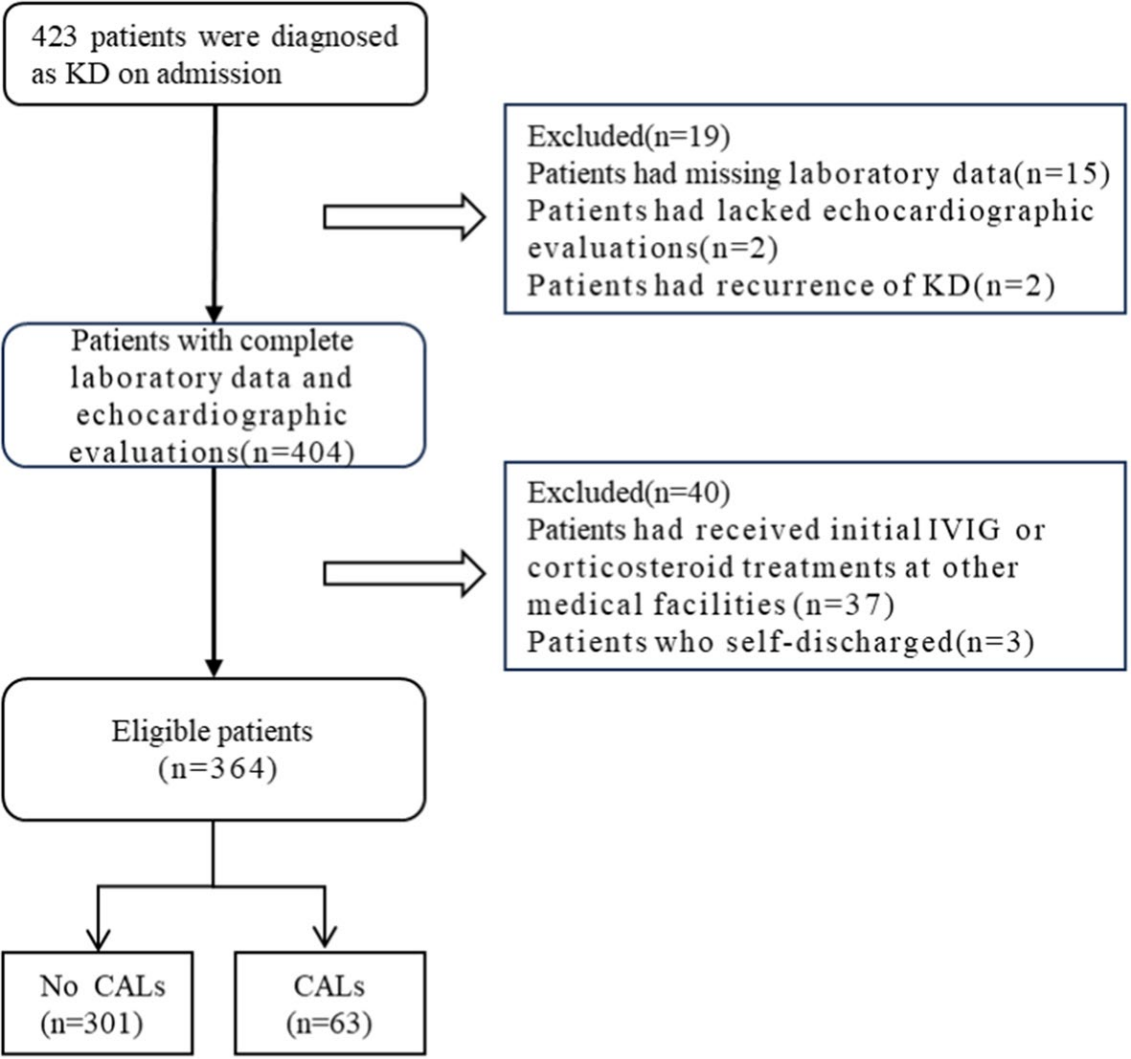
Flowchart of our research. *KD* Kawasaki disease, *IVIG* intravenous immunoglobulin, *CALs* coronary artery lesions

Over the course of the study, our institution managed 423 patients initially diagnosed with KD. Of these, 15 lacked crucial laboratory data, and 2 did not undergo echocardiographic evaluations at admission, 2 had recurrent KD episodes. Of the remaining 404 patients, 40 were further excluded for the following reasons: 37 were excluded because they had received their initial IVIG or corticosteroid treatments at different medical facilities, and 3 due to self-discharge. (Fig. 1).

### Data collection

We compiled detailed data on pediatric patients diagnosed with KD, encompassing demographics (such as gender and age), clinical indicators (like disease duration at IVIG therapy onset, IKD), laboratory parameters, and CALs metrics upon admission. The laboratory data were derived from initial blood tests performed at the time of admission for KD-diagnosed patients. This included White Blood Cell (WBC) counts, Neutrophil (NEU) counts, Platelet (PLT) counts, Lymphocyte (LYM) counts, Red Cell Distribution Width (RDW), Hemoglobin (Hb) concentrations, Mean Platelet Volume (MPV), C-reactive protein (CRP) concentrations, Serum Albumin (ALB) levels, Alanine aminotransferase (ALT), Aspartate aminotransferase (AST), Erythrocyte Sedimentation Rate (ESR), and Serum Sodium (Na) concentrations. We used these to calculate the Neutrophil-to-Lymphocyte Ratio (NLR, represented as NEU/LYM) and the Mean Platelet Volume-to-Lymphocyte Ratio (MPVLR, represented as MPV/LYM). The Systemic Immune-Inflammation Index (SII) was ascertained using the formula: SII = PLT × NEU/LYM. Information concerning CALs was sourced from the initial echocardiographic evaluations during the admission process.

#### Methods of examination and instrumentation

Upon admission, all KD patients undergo the collection of 2 mL of peripheral venous blood using an EDTA anticoagulant tube. The collected blood is meticulously mixed. Subsequently, an automated hematology analyzer, specifically the SYSMEX XE-2100 (F5192), is employed for the comprehensive analysis of blood parameters, which include but are not limited to WBC, NEU, LYM, PLT, and various others. Concurrently, a separate collection of 3 mL of venous blood occurs using a separation gel tube. This blood specimen is destined for the analysis of markers such as ALT, AST, ALB, CRP, Na, and other biochemical indicators, facilitated by the Cobas 6000 automated biochemical analyzer. The evaluation of the ESR involves the collection of 2 mL of blood into a sodium citrate anticoagulant tube. Following the collection, a reverse-shaking procedure is executed, and the ESR is quantified using the Westergren method. Cardiac ultrasound examinations are executed by specialized physicians hailing from the Cardiac Ultrasonography Department at the First Affiliated Hospital of Anhui Medical University. These examinations are performed using the Philips EPIQ 7C ultrasound machine for a comprehensive evaluation.

### Definitions

The criteria for CALs are defined as follows: The luminal diameter of the coronary artery trunk should be ≤ 2.5 mm for individuals aged ≤ 3 years, ≥ 3 mm for those aged 4–9 years, and ≥ 3.5 mm for those aged 10–14 years, as specified by recognized guidelines [15]; If a patient exhibits a persistent or recurrent fever above 38.0 °C for over 36 h following the initiation of IVIG therapy, there exists a notable concern for IVIG resistance^1^. Administering IVIG treatment beyond the 10th day of the illness is referred to as “delayed IVIG therapy”.

### Statistical analysis

Quantitative data are presented as either mean ± standard deviation ($\overline{x }$± s)or median (interquartile range). Intergroup comparisons for these data types utilize either independent sample *t* tests or Mann–Whitney *U* tests. Categorical data, on the other hand, are expressed as percentages, with intergroup comparisons assessed using *χ*2 tests. The binary logistic regression analysis aids in discerning risk factors for CALs at admission, while multivariate adjusted analysis gauges the association between SII and CALs. Three distinct models were devised to understand the relationship between SII and CALs upon admission. Model 1 was unadjusted; Model 2 accounted for age and delayed treatment; Model 3 made additional adjustments for albumin and serum sodium beyond those in Model 2. To detect any linear association between SII and CALs, restricted cubic spline tests (RCS) were applied within a binary logistic regression framework. Additionally, a curve fitting analysis was undertaken for SII values at admission against the presence of CALs. The Spearman correlation analysis evaluated the possible association between C-reactive protein (CRP) and SII. To ascertain and juxtapose the predictive prowess of variables for CALs at admission, both receiver operating characteristic (ROC) curves and the area under the curve (AUC) were deployed. Subgroup analyses assessed variables such as gender, IVIG resistance, IKD, and the timing since IVIG treatment commencement (< 10 days vs. ≥ 10 days). All statistical deliberations were facilitated using R for Windows (Version 4.3.1) and SPSS 27.0 (SPSS Inc. Chicago, IL, USA). A significance level of *P* < 0.05 was considered indicative of statistical significance.

## Results

### Clinical characteristics of KD patients

Of the 364 pediatric patients enrolled, 206 were males (56.6%) and 158 were females (43.4%), with a median age of 25 months. Clinical characteristics of these children were documented prior to the initiation of IVIG therapy (Table 1). Out of the 364 cases, 26 (7.1%) were diagnosed with Incomplete Kawasaki disease (IKD). Sixty-three patients exhibited CALs upon admission. These patients tended to be younger, were more likely to receive delayed IVIG treatment, and presented elevated counts of white blood cells, neutrophils, and platelets, along with increased SII and CRP levels. In contrast, they displayed lower lymphocyte ratios and decreased concentrations of albumin and serum sodium (*P* < 0.05).

**Table 1:** Characteristics of all patients

Variables	No CALs (*n* = 301)	CALs (*n* = 63)	*P*
Age (mon), median (quartile)	26.0 (15.0–41.0)	20.0 (11.0–35.0)	0.048
Sex, *n* (%)			0.224
Male	166 (55.1)	40 (63.5)	
Female	135 (44.9)	23 (36.5)	
IKD, *n* (%)			0.788
Yes	21 (7.0)	5 (7.9)	
No	280 (93.0)	58 (92.1)	
Days of IVIG at initiation,mean ± SD	6.30 ± 0.96	7.41 ± 2.37	< 0.001
Days of IVIG at initiation, *n* (%)			< 0.001
< 10	297 (98.7)	50 (79.4)	
≥ 10	4 (1.3)	13 (20.6)	
WBC (× 10^9^/L), mean ± SD	13.87 ± 4.82	16.66 ± 4.77	< 0.001
NEU (× 10^9^/L), mean ± SD	9.17 ± 4.23	11.49 ± 4.68	< 0.001
NEU (%), mean ± SD	65.14 ± 14.32	67.98 ± 14.52	0.160
LYM (× 10^9^/L), mean ± SD	3.53 ± 1.89	3.64 ± 1.99	0.675
LYM (%), mean ± SD	26.74 ± 12.19	22.67 ± 11.33	0.012
Hb (g/L), mean ± SD	115.03 ± 67.37	110.51 ± 11.71	0.596
RDW (%), mean ± SD	13.30 ± 1.19	13.28 ± 0.79	0.886
PLT (× 10^9^/L), median (quartile)	342.00 (283.00–427.00)	375.00 (312.00–516.00)	0.030
MPV (fL), mean ± SD	9.92 ± 0.87	10.02 ± 0.89	0.459
NLR, median (quartile)	2.57 (1.51–4.60)	2.67 (1.77–6.52)	0.065
MPVLR, median (quartile)	3.10 (2.14–4.48)	2.86 (1.90–4.95)	0.746
SII, median (quartile)	887.96 (519.66–1504.12)	1128.74 (720.09–2399.13)	0.002
ALT (u/L), median (quartile)	29.00 (18.00–63.00)	27.00 (15.00–44.00)	0.176
AST (u/L), median (quartile)	29 (23.00–43.25)	30.00 (24.00–42.00)	0.706
ALB (g/L), median (quartile)	38.00 (35.45–40.85)	34.00 (30.00–40.00)	< 0.001
ESR (mm/h), mean ± SD	60.40 ± 21.07	60.37 ± 22.74	0.991
Na (mmol/L), mean ± SD	136.29 ± 3.41	135.25 ± 3.17	0.026
CRP (mg/L), median (quartile)	61.04 (31.03,97.58)	83.16 (54.84,103.34)	< 0.001

*CALs* coronary artery lesions, *KD* Kawasaki disease, *IVIG* intravenous immunoglobulin, *WBC* white blood cells, *NEU* neutrophil, *LYM* lymphocyte, *Hb* hemoglobin, *RDW* red cell distribution width, *PLT* platelet, *MPV* mean platelet volume, *NLR* neutrophil-to-lymphocyte ratio, *MPVLR* Mean Platelet Volume-to-lymphocyte ratio, *SII* the Systemic Immune-Inflammatory Index, *ALT* alanine aminotransferase, *AST* aspartate aminotransferase, *ALB* serum albumin, *ESR* erythrocyte sedimentation rate, *Na* serum sodium, *CRP* C-reactive protein

### Binary logistic regression modeling analysis was employed to predict the occurrence of CALs upon admission

Table 2 presents the risk factors for CALs as determined through binary logistic regression analysis. Individual risk factors for the emergence of CALs upon admission encompass younger age, delayed treatment, elevated white blood cell count, increased platelet count, heightened SII and CRP levels, complemented by decreased levels of albumin and serum sodium. The relationship between SII and the onset of CALs upon admission, both adjusted and unadjusted for various factors, is outlined (Table 3). In Model 1, without any adjustments, a pronounced positive relationship between SII and CALs at the point of admission was observed (OR = 1.003, 95%CI 1.001–1.007, *P* < 0.001). In subsequent models, both Model 2 (OR = 1.002, 95%CI 1.002–1.005) and Model 3 (OR = 1.003, 95%CI 1.003–1.010), after partial and full adjustments for potential confounders, respectively, consistently demonstrated that a higher SII is a significant risk indicator for CALs upon admission (*P* < 0.001).

**Table 2:** Binary logistic regression analysis to evaluate risk factors for CALs on admission

Variables	*B*	S.E	Waldχ2		OR (95%CI)	*P*
Age (mon)	− 0.017	0.008		4.227	0.983 (0.967,0.999)	0.040
Days of IVIG at initiation	0.497	0.105		22.358	1.644 (1.338,2.021)	< 0.001
Days of IVIG at initiation, *n* (%)						
< 10	− 2.960	0.592		25.019	0.052 (0.016,0.165)	< 0.001
≥ 10	2.960	0.592		25.019	19.305 (6.052,61.582)	< 0.001
WBC (× 10^9^/L)	0.074	0.060		1.494	1.076 (0.957,1.211)	0.222
NEU (× 10^9^/L)	0.031	0.065		0.225	1.031 (0.908,1.170)	0.635
PLT (× 10^9^/L)	0.004	0.001		10.210	1.004 (1.001,1.006)	0.001
SII	0.003	0.001		64.189	1.003 (1.001,1.007)	< 0.001
ALB (g/L)	− 0.097	0.029		11.034	0.908 (0.858,0.961)	< 0.001
Na (mmol/L)	− 0.092	0.042		4.867	0.912 (0.840,0.990)	0.027
CRP (mg/L)	0.010	0.003		11.290	1.010 (1.004,1.016)	< 0.001

*IVIG* intravenous immunoglobulin, *WBC* white blood cells, *NEU* neutrophil, *PLT* platelet, *SII* the Systemic Immune-Inflammatory Index, *ALB* serum albumin, *ESR* erythrocyte sedimentation rate, *Na* serum sodium

**Table 3:** Relationship between SII and CALs on admission in different models

Models	SII [OR (95%CI)]	*P*
Model 1 Model 2 Model 3	1.003 (1.001,1.007) 1.002 (1.002,1.005) 1.004 (1.003,1.010)	< 0.001 < 0.001 < 0.001

Model 1: Unadjusted; Model 2: Adjusted for sex and delayed IVIG treatment post the 10th day; Model 3: Adjusted based on Model 2, with additional adjustments for albumin and serum sodium

### The relationship between SII and CALs at admission

**Fig. 2:**
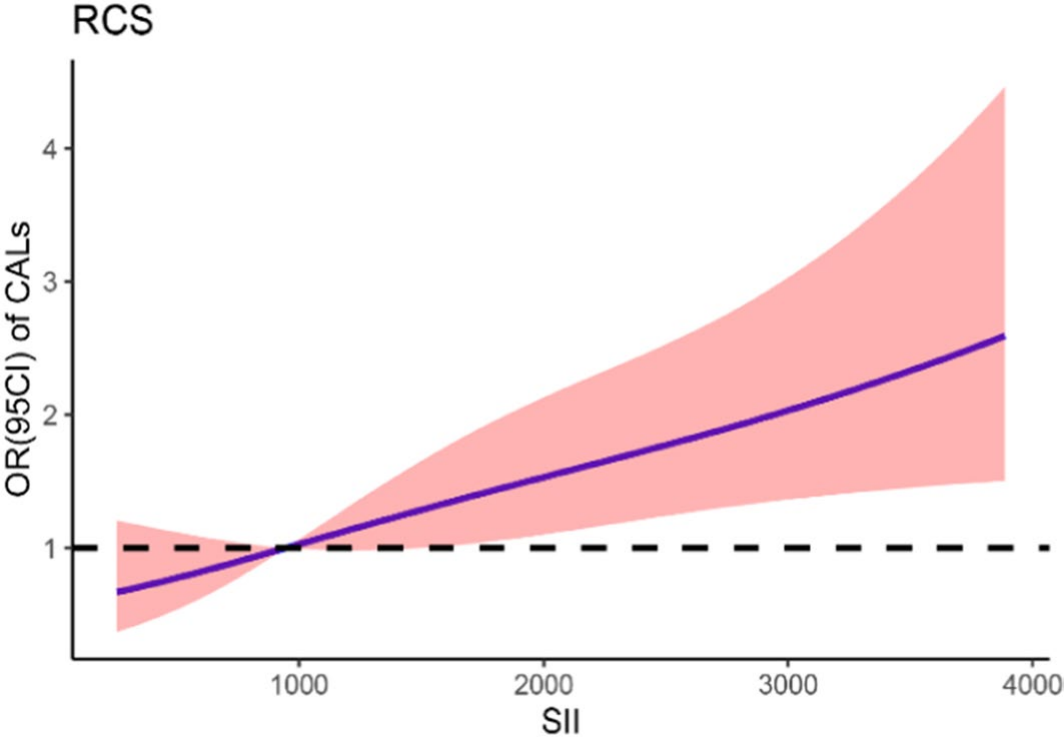
The adjusted odds ratio (OR) represents the relationship between SII and CALs upon admission. The solid line delineates the OR, while the shaded region signifies the 95% confidence interval (95%CI). *CALs* coronary artery lesions, *SII* the Systemic Immune-Inflammatory Index

**Fig. 3:**
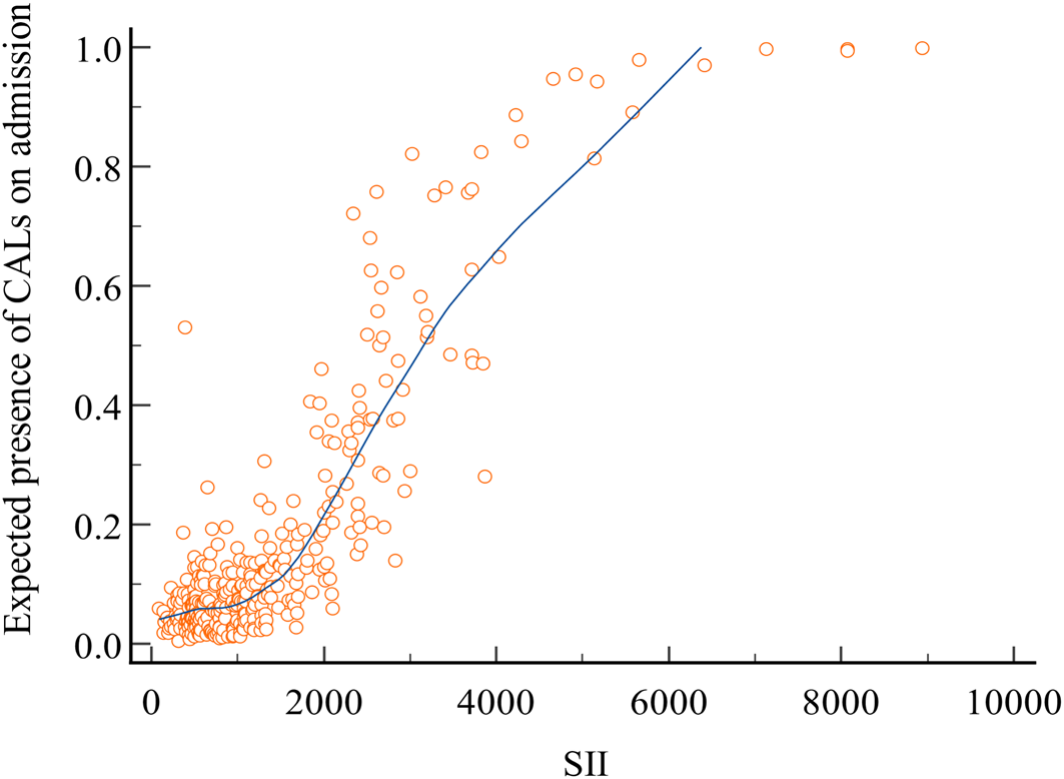
A fitting model was utilized to predict the relationship between SII and the anticipated presence of CALs upon admission. The circles symbolize SII values and their associated expectations. Concurrently, the actual presence of CALs at the time of admission is represented by a line, which serves as the fitting curve based on observed results

Using the restricted cubic spline (RCS) test method and after adjusting for confounding factors, a notable positive linear relationship was observed between SII and the odds ratio of CALs (*P* for nonlinearity was 0.54, Fig. 2). Furthermore, after adjusting for other confounders, there was a positive correlation between the predicted presence of CALs upon admission and SII (Fig. 3).

### The relationship between SII and CRP, as well as the ROC curve analysis for both in predicting the occurrence of CALs upon admission

**Fig. 4:**
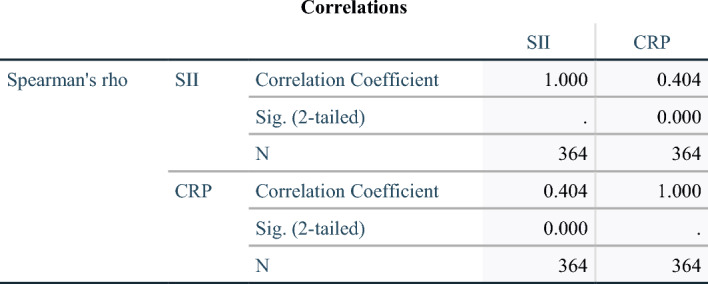
This suggests an exploration into the relationship between CRP and SII in patients with KD. *CRP* C-reactive protein, *SII* the Systemic Immune-Inflammation Index

**Fig. 5:**
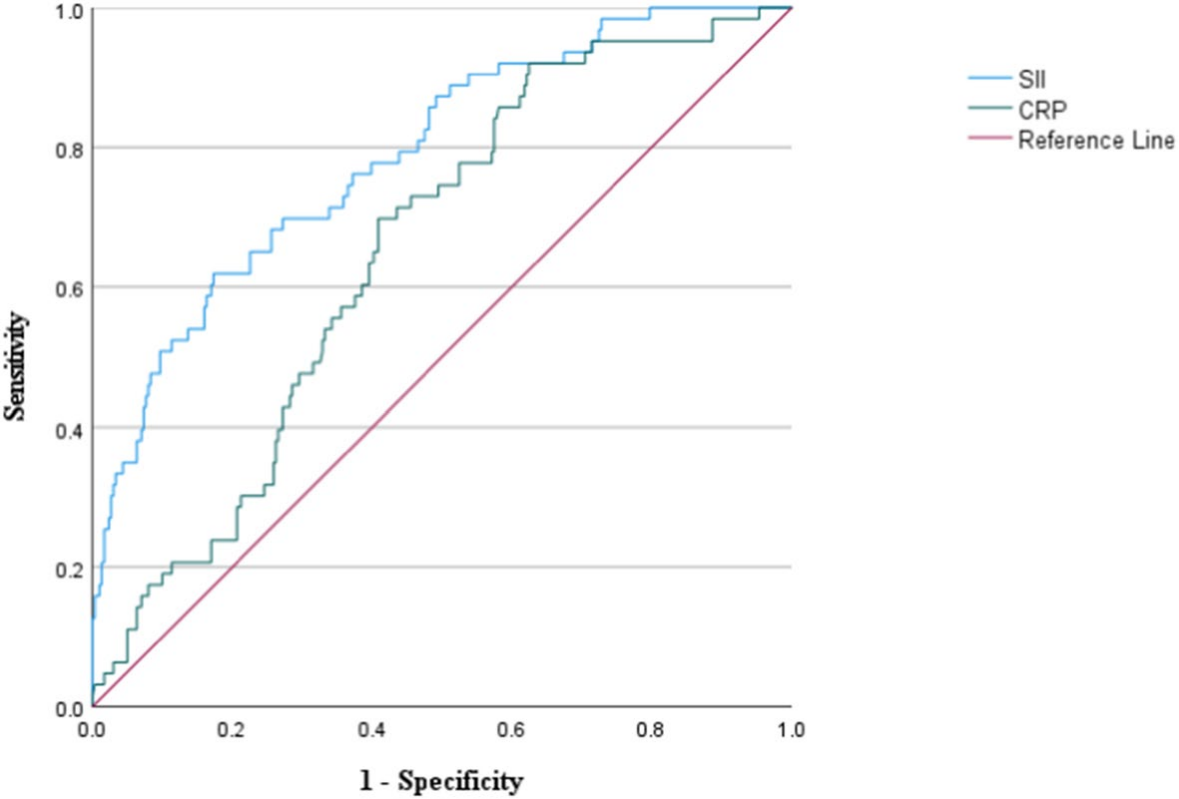
The ROC curve for SII and CRP in predicting CALs; *CALs* coronary artery lesions, *SII* the Systemic Immune-Inflammatory Index, *CRP* C-reactive protein

As unveiled by Spearman correlation analysis, there is a positive correlation between SII and CRP levels (*r* = 0.404, *P* < 0.001, Fig. 4). Analyzing via the ROC curve revealed that the SII's predictive power for CALs upon admission achieved an Area Under the Curve (AUC) value of 0.789 (95%CI 0.723–0.855, *P* < 0.001, Fig. 5), and the CRP's predictive power for CALs upon admission achieved an AUC value of 0.652 (95%CI 0.585–0.718, *P* < 0.001, Fig. 5).

## Discussion

As widely recognized in the medical community, CALs stand as the paramount complication inherent to KD. In our comprehensive study, it becomes unequivocally manifest that SII emerges as a pivotal determinant in assessing the risk for the inception of CALs during the initial phase of admission. An unmistakable linear correlation between SII and CALs is evident, accentuating the profound prognostic value of SII in anticipating the manifestation of CALs.

Research consistently indicates that KD is an acute, self-limited, systemic vasculitis, with the immune response and associated inflammatory processes central to its pathogenesis [16]. This disease affects a range of small to medium-sized arteries, impacting various organs such as the kidneys, lungs, and coronary vessels [17]. During the acute phase of KD, we observe not only increased levels of standard inflammatory markers, including WBC and CRP [1], but also a rise in pro-inflammatory cytokines like interleukin-10 (IL-10) and tumor necrosis factor alpha (TNF-ɑ) [18].

Neutrophils, among the most prominent leukocytes in the human circulatory system, play a crucial role in the innate cellular immune response, significantly influencing the regulation of inflammatory reactions [19]. In conditions like atherosclerosis, neutrophils can intensify tissue damage and inflammation by promoting the breakdown and death of smooth muscle cells [19]. Research suggests that when faced with microbial threats, activated neutrophils quickly move through the bloodstream to the sites of inflammation. Here, they perform various tasks such as phagocytosis, degranulation, respiratory burst, and the formation of neutrophil extracellular traps (NETs); all aimed at neutralizing pathogenic microorganisms [20]. Furthermore, neutrophils are recognized as early indicators of the pro-inflammatory cytokine environment during thrombus formation [21, 22]. During the acute phase of KD, there is an observed increase in neutrophil count, and their functionality is heightened with the release of reactive oxygen species, neutrophil elastase, and myeloperoxidase. This increased activity may lead to tissue damage. Present understanding suggests that the damage to endothelial cells caused by activated neutrophils contributes to the development of KD vasculitis [23, 24].

Lymphocytes, a prominent subclass of leukocytes, function distinctly from neutrophils, providing them with anti-inflammatory properties critical for maintaining the health of the vascular endothelium. Animal studies have shown that lymphocytes are involved in modulating inflammatory responses [25]. Prior research has demonstrated a relationship between low lymphocyte counts and a higher occurrence of cardiovascular diseases [26]. Specifically, reduced lymphocyte levels have been associated with the accelerated progression of atherosclerosis [27]. Moreover, a decreased lymphocyte count has been correlated with poor outcomes in several cardiovascular conditions, including acute coronary syndrome [28] and heart failure [29].

Platelets, small disk-shaped cell fragments characterized by their lack of a cellular nucleus, originate within the bone marrow from megakaryocytes, large precursor cells. These megakaryocytes undergo a process known as cytoplasmic fragmentation, which results in the formation of numerous platelets. Once formed, these platelets are released into the bloodstream, where they play a vital role in hemostasis, blood clotting, and the inflammatory response [30].They circulate in the blood for a lifespan ranging from 7 to 10 days before being cleared by the spleen and liver [31]. In the context of arterial thrombosis formation, platelets play a crucial role. In addition to their function in thrombus formation, platelets also participate in inflammatory responses and contribute to the progression of atherosclerosis [32]. Inflammation can stimulate platelet aggregation and their adherence to endothelial cell surfaces, resulting in tissue ischemia, hypoxia, microthrombi formation, and ultimately, tissue necrosis [33]. Elevated platelet counts have been observed in KD patients, usually appearing between the 2nd to 3rd week of the disease [1, 31]. Initially, this increase was considered a benign and reactive response. However, recent research indicates that it is not just a quantitative rise in platelets; these cells also demonstrate heightened adherence within blood vessels and exhibit activation. Upon activation, platelets can release a spectrum of substances that encourage vasoconstriction and coagulation, thereby increasing the risk of coronary artery thrombosis and potential infarction [34–36].

Based on the aforementioned discussions, SII—a novel inflammatory marker that integrates neutrophils, lymphocytes, and platelets—appears promising as a more effective predictor than its individual components alone. Some researchers argue that the SII provides a more holistic and balanced evaluation of the body's immune response and inflammation dynamics [37]. Evidence shows that SII is associated with negative outcomes in heart failure [12]. Moreover, various studies have highlighted its significance in forecasting adverse cardiovascular events [38]. Therefore, while the SII can act as an auxiliary laboratory measure for predicting CALs, it should not be exclusively depended upon as a predictive marker for CALs progression. The robust correlation between SII and CRP indicates that KD patients with elevated SII levels may experience an increased risk of intense inflammatory responses during the acute phase.

We observed a linear relationship between SII values upon admission and the onset of CALs. A clear positive correlation between SII and CALs suggests that higher SII levels could signify a heightened risk for CALs development. Of the three SII components, lymphocyte count did not exhibit significant differences between KD patients with and without CALs. Nonetheless, the percentage of lymphocytes was lower in the CALs group. This indicates that lymphocyte levels are diminished in patients who present with CALs. The surge in SII is attributed to the increased counts of neutrophils and platelets. We posit that a rise in these components intensifies the immune-inflammatory response, potentially exacerbating the disease progression in KD patients after the illness onset.

Our study is not without limitations. First, the retrospective nature and single-center setup could introduce inherent selection biases. Second, our primary focus was on CALs evident at KD admission since most CALs manifest during the early stages and typically recede as the disease progresses. Third, due to sample size restrictions, we did not analyze IVIG-resistant cases. Finally, the SII serves as an indicator for initial CALs upon admission and not for progressive CALs; Hence, a high SII shouldn't be construed as a call for more aggressive treatments. Future research should target larger cohorts and adopt multi-center, prospective methodologies.

## References

[1] McCrindle BW, Rowley AH, Newburger JW, et al. Diagnosis, treatment, and long-term management of Kawasaki disease: a scientific statement for health professionals from the American Heart Association. Circulation. 2017;135(17):e927–99. 28356445 10.1161/CIR.0000000000000484

[2] Suzuki A, Kamiya T, Kuwahara N, et al. Coronary arterial lesions of Kawasaki disease: cardiac catheterization findings of 1100 cases. Pediatr Cardiol. 1986;7(1):3–9. 3774580 10.1007/BF02315475

[3] Miura M, Kobayashi T, Kaneko T, et al. Association of severity of coronary artery aneurysms in patients with Kawasaki disease and risk of later coronary events. JAMA Pediatr. 2018;172(5):e180030. 29507955 10.1001/jamapediatrics.2018.0030 PMC5875323

[4] Kuwabara M, Yashiro M, Kotani K, et al. Cardiac lesions and initial laboratory data in Kawasaki disease: a nationwide survey in Japan. J Epidemiol. 2015;25(3):189–93. 25716055 10.2188/jea.JE20140128 PMC4340995

[5] Tacke CE, Breunis WB, Pereira RR, Breur JM, Kuipers IM, Kuijpers TW. Five years of Kawasaki disease in the Netherlands: a national surveillance study. Pediatr Infect Dis J. 2014;33(8):793–7. 24463809 10.1097/INF.0000000000000271

[6] Ishihara H, Izumida N, Hosaki J. Criterion for early prediction of coronary artery involvement by clinical manifestations in patients with Kawasaki disease. Bull Tokyo Med Dent Univ. 1985;32(2):77–89. 3864563

[7] Koyanagi H, Nakamura Y, Yanagawa H. Lower level of serum potassium and higher level of C-reactive protein as an independent risk factor for giant aneurysms in Kawasaki disease. Acta Paediatr. 1998;87(1):32–6. 9510444 10.1080/08035259850157831

[8] Marrani E, Burns JC, Cimaz R. How should we classify Kawasaki disease? Front Immunol. 2018;9:2974. 30619331 10.3389/fimmu.2018.02974 PMC6302019

[9] Hu B, Yang XR, Xu Y, et al. Systemic immune-inflammation index predicts prognosis of patients after curative resection for hepatocellular carcinoma. Clin Cancer Res. 2014;20(23):6212–22. 25271081 10.1158/1078-0432.CCR-14-0442

[10] Wang BL, Tian L, Gao XH, et al. Dynamic change of the systemic immune inflammation index predicts the prognosis of patients with hepatocellular carcinoma after curative resection. Clin Chem Lab Med. 2016;54(12):1963–9. 27010778 10.1515/cclm-2015-1191

[11] Dolan RD, McSorley ST, Park JH, et al. The prognostic value of systemic inflammation in patients undergoing surgery for colon cancer: comparison of composite ratios and cumulative scores. Br J Cancer. 2018;119(1):40–51. 29789606 10.1038/s41416-018-0095-9 PMC6035216

[12] Yang R, Chang Q, Meng X, Gao N, Wang W. Prognostic value of Systemic immune-inflammation index in cancer: a meta-analysis. J Cancer. 2018;9(18):3295–302. 30271489 10.7150/jca.25691 PMC6160683

[13] Xu M, Chen R, Liu L, et al. Systemic immune-inflammation index and incident cardiovascular diseases among middle-aged and elderly Chinese adults: The Dongfeng-Tongji cohort study. Atherosclerosis. 2021;323:20–9. 33773161 10.1016/j.atherosclerosis.2021.02.012

[14] Jin Z, Wu Q, Chen S, et al. The associations of two novel inflammation indexes, SII and SIRI with the risks for cardiovascular diseases and all-cause mortality: a ten-year follow-up study in 85,154 individuals. J Inflamm Res. 2021;14:131–40. 33500649 10.2147/JIR.S283835 PMC7822090

[15] Kobayashi T, Ayusawa M, Suzuki H, et al. Revision of diagnostic guidelines for Kawasaki disease (6th revised edition). Pediatr Int. 2020;62(10):1135–8. 33001522 10.1111/ped.14326

[16] Lindquist ME, Hicar MD. B cells and antibodies in Kawasaki disease. Int J Mol Sci. 2019;20(8). 10.3390/ijms20081834 PMC6514959 31013925

[17] Sakurai Y. Autoimmune aspects of Kawasaki disease. J Investig Allergol Clin Immunol. 2019;29(4):251–61. 30183655 10.18176/jiaci.0300

[18] Mahmoudinezhad Dezfouli SM, Salehi S, Khosravi S. Pathogenic and therapeutic roles of cytokines in Kawasaki diseases. Clin Chim Acta. 2022;532:21–8. 35609708 10.1016/j.cca.2022.05.015

[19] Fernández-Ruiz I. Neutrophil-driven SMC death destabilizes atherosclerotic plaques. Nat Rev Cardiol. 2019;16(8):455. 31101895 10.1038/s41569-019-0214-1

[20] Papayannopoulos V. Neutrophil extracellular traps in immunity and disease. Nat Rev Immunol. 2018;18(2):134–47. 28990587 10.1038/nri.2017.105

[21] Darbousset R, Thomas GM, Mezouar S, et al. Tissue factor-positive neutrophils bind to injured endothelial wall and initiate thrombus formation. Blood. 2012;120(10):2133–43. 22837532 10.1182/blood-2012-06-437772

[22] von Brühl ML, Stark K, Steinhart A, et al. Monocytes, neutrophils, and platelets cooperate to initiate and propagate venous thrombosis in mice in vivo. J Exp Med. 2012;209(4):819–35. 22451716 10.1084/jem.20112322 PMC3328366

[23] Biezeveld MH, van Mierlo G, Lutter R, et al. Sustained activation of neutrophils in the course of Kawasaki disease: an association with matrix metalloproteinases. Clin Exp Immunol. 2005;141(1):183–8. 15958085 10.1111/j.1365-2249.2005.02829.x PMC1809423

[24] Hartman CL, Ford DA. MPO (myeloperoxidase) caused endothelial dysfunction. Arterioscler Thromb Vasc Biol. 2018;38(8):1676–7. 30354198 10.1161/ATVBAHA.118.311427 PMC6324573

[25] Jander S, Kraemer M, Schroeter M, Witte OW, Stoll G. Lymphocytic infiltration and expression of intercellular adhesion molecule-1 in photochemically induced ischemia of the rat cortex. J Cereb Blood Flow Metab. 1995;15(1):42–51. 7528223 10.1038/jcbfm.1995.5

[26] Horne BD, Anderson JL, John JM, et al. Which white blood cell subtypes predict increased cardiovascular risk? J Am Coll Cardiol. 2005;45(10):1638–43. 15893180 10.1016/j.jacc.2005.02.054

[27] Núñez J, Miñana G, Bodí V, et al. Low lymphocyte count and cardiovascular diseases. Curr Med Chem. 2011;18(21):3226–33. 21671854 10.2174/092986711796391633

[28] Núñez J, Núñez E, Bodí V, et al. Low lymphocyte count in acute phase of ST-segment elevation myocardial infarction predicts long-term recurrent myocardial infarction. Coron Artery Dis. 2010;21(1):1–7. 20050312 10.1097/mca.0b013e328332ee15

[29] Levy WC, Mozaffarian D, Linker DT, et al. The Seattle Heart Failure Model: prediction of survival in heart failure. Circulation. 2006;113(11):1424–33. 16534009 10.1161/CIRCULATIONAHA.105.584102

[30] Zarbock A, Polanowska-Grabowska RK, Ley K. Platelet-neutrophil-interactions: linking hemostasis and inflammation. Blood Rev. 2007;21(2):99–111. 16987572 10.1016/j.blre.2006.06.001

[31] Quach ME, Chen W, Li R. Mechanisms of platelet clearance and translation to improve platelet storage. Blood. 2018;131(14):1512–21. 29475962 10.1182/blood-2017-08-743229 PMC5887765

[32] Pasalic L, Wang SS, Chen VM. Platelets as biomarkers of coronary artery disease. Semin Thromb Hemost. 2016;42(3):223–33. 26926585 10.1055/s-0036-1572328

[33] von Ungern-Sternberg SNI, Vogel S, Walker-Allgaier B, et al. Extracellular cyclophilin a augments platelet-dependent thrombosis and thromboinflammation. Thromb Haemost. 2017;117(11):2063–78. 28981554 10.1160/TH17-01-0067 PMC5885247

[34] Laurito M, Stazi A, Delogu AB, et al. Endothelial and platelet function in children with previous Kawasaki disease. Angiology. 2014;65(8):716–22. 24019084 10.1177/0003319713502392

[35] Jin J, Wang J, Lu Y, et al. Platelet-derived microparticles: a new index of monitoring platelet activation and inflammation in Kawasaki disease. Indian J Pediatr. 2019;86(3):250–5. 30159809 10.1007/s12098-018-2765-2

[36] Kim HJ, Choi EH, Lim YJ, Kil HR. The usefulness of platelet-derived microparticle as biomarker of antiplatelet therapy in Kawasaki disease. J Korean Med Sci. 2017;32(7):1147–53. 28581272 10.3346/jkms.2017.32.7.1147 PMC5461319

[37] Fest J, Ruiter R, Ikram MA, Voortman T, van Eijck CHJ, Stricker BH. Reference values for white blood-cell-based inflammatory markers in the Rotterdam Study: a population-based prospective cohort study. Sci Rep. 2018;8(1):10566. 30002404 10.1038/s41598-018-28646-w PMC6043609

[38] Yang YL, Wu CH, Hsu PF, et al. Systemic immune-inflammation index (SII) predicted clinical outcome in patients with coronary artery disease. Eur J Clin Invest. 2020;50(5): e13230. 32291748 10.1111/eci.13230

